# Molecular evidence depicts genetic divergence among *Agropyron elongatum* and *A*. *cristatum* accessions from gene pool of Iran

**DOI:** 10.1371/journal.pone.0294694

**Published:** 2023-11-30

**Authors:** Hamid Hatami Maleki, Reza Mohammadi, Fatemeh Firouzkuhi, Reza Darvishzadeh, Hossein Zeinalzadeh-Tabrizi

**Affiliations:** 1 Department of Plant Production and Genetics, Faculty of Agriculture, University of Maragheh, Maragheh, Iran; 2 Branch for Northwest & West Region, Agricultural Biotechnology Research Institute of Iran (ABRII), Agricultural Research, Education and Extension Organization (AREEO), Tabriz, Iran; 3 Department of Plant Production and Genetics, Faculty of Agriculture, Urmia University, Urmia, Iran; 4 Department of Horticulture and Agronomy, Faculty of Agriculture, Kyrgyz-Turkish Manas University, Bishkek, Kyrgyzstan; Institute of Genetics and Developmental Biology Chinese Academy of Sciences, CHINA

## Abstract

The genus *Agropyron* has an important role in soil protection and forage production in rangelands. The investigation utilized 37 ISSR primers, resulting in the detection of 956 loci within the *A*. *elongatum* genome and 705 loci within the *A*. *cristatum* genome. The findings revealed a high level of polymorphism, with 97% of loci in *A*. *elongatum* and 84% of loci in *A*. *cristatum* exhibiting variability. Notably, the primer (AC)_8_GCT emerged as a promising candidate for evaluating genetic diversity due to its ability to amplify numerous loci in both species. Using both the UPGMA algorithm and Bayesian analysis, the examined *Agropyron* accessions were categorized into two subgroups based on their respective species. The Q values associated with these subgroups suggested that certain accessions, namely "G16," "G19," "G20," "G21," "G22," "G23," "G24," and "G25," displayed potential admixture genomes. An analysis of molecular variance (AMOVA) underscored the significance of within-species variability, which accounted for 69% of the overall diversity, compared to between-species variability at 31%. Various genetic diversity parameters, including Na, Ne, I, He, and the number of private loci, were found to be higher in *A*. *elongatum* when compared to *A*. *cristatum*. Furthermore, Jaccard similarity coefficients ranged from 0.33 to 0.66 in *A*. *cristatum* and from 0.25 to 0.7 in *A*. *elongatum*, indicating the extent of genetic relatedness among these species. Intriguingly, the study identified two and three heterotic groups in *A*. *cristatum* and *A*. *elongatum*, respectively, which could be harnessed in the development of synthetic varieties to exploit heterosis. The results also indicated that a small proportion of ISSR loci pairs (5.2% in *A*. *elongatum* and 0.5% in *A*. *cristatum*) exhibited significant levels of linkage disequilibrium (LD) (P≤0.05), suggesting the potential utility of LD-based association mapping in *Agropyron* species. In conclusion, this research sheds light on the genetic diversity of *Agropyron* species and provides valuable insights into their potential applications in soil protection and forage production, as well as the prospects for enhancing genetic variability and heterosis in these species.

## Introduction

The genus *Agropyron*, from the family Poaceae, is one of the important pasture plants that is native to Europe and Asia. *Agropyron* spp. is a perennial herb with diploid (2n = 2x = 14), tetraploid (2n = 4x = 28), and hexaploid (2n = 6x = 42) genomic formulas [1]. It is shown to be a good supply of animal feed as well as a suitable habitat for household animals and wildlife. These genus’ plants are useful for weed control, soil stability, and watershed management, as well as providing a rich gene pool for breeding bread wheat varieties [2, 3]. Moreover, *Agropyron* plants have beneficial traits such as resistance to biotic and abiotic challenges such as low temperature, salt, drought, diseases, and pests, which protect the plant’s gene pool and lead to enhanced pastures and greater fodder production [4, 5].

The genus *Agropyron* contains several species, 19 of which are found in Iran [6]. The identified species of Agropyron are allopolyploids with the P genome. This genome can be combined with the St genome from *Pseudoroegneria spicata* and the Y genome from an unknown donor in various allopolyploid combinations, including the StP genome from *Douglasdeweya wangii* and the StYP genome from the genus *Kengyilia* [7]. *A*. *cristatum* (2n = 28, genome PPPP), known as crested wheatgrass, is a perennial plant with medium height that is known as a cool-season plant and is resistant to drought as well as cold stresses [4]. *A*. *cristatum* has a divergent and deep root system, which makes it a suitable choice for soil stabilization. There are some reports about crossing *A*. *cristatum* with bread or durum wheat with the aim of introducing desirable genes like disease resistance as well as grain yield from *A*. *cristatum*. By acquiring wheat-*A*. *cristatum* disomic addition lines, it is possible to utilize these desired genes for improving wheat varieties. Till now, wheat-*A*. *cristatum* 1P, 2P, 3P, 4P, 6P, and 7P disomic addition lines have been successfully created, and many excellent genes were located in specific chromosomes and transmitted into wheat [8]. For example, it has been found that the *A*. *cristatum* 6P addition line confers gene clusters related to yield, such as multiple florets and grains per spike, and the 2P addition line possesses gene clusters related to disease resistance, including powdery mildew, leaf rust, and stripe rust [9–11]. Nevertheless, it’s worth noting that the wheat-*A*. *cristatum* 5P disomic addition line has not been documented. Another notable species belonging to the *Agropyron* genus is *A*. *elongatum*, commonly referred to as tall wheatgrass, renowned for its robust resistance to salinity stress and its ability to endure challenging environmental conditions [5]. It is an essential natural source for wheat breeding because of its high seed protein content and resilience to plant disease. Many different types of hybrid plants have been developed by sexually crossing common wheat with *A*. *elongatum* or *A*. *cristatum* [5, 12]. Disease resistance genes, along with traits related to plant characteristics like dwarfism, photosynthetic efficiency, and yield, have been effectively incorporated into wheat from *A*. *elongatum*. As a result, several valuable wheat genotypes have been released, including Xiaoyan 6, Xiaoyan 759, Xiaoyan 22, Gaoyou 504, and Shanrong 3 in China, as well as Oasis and Seri in Mexico [3]. In detail, *Lr24/Sr24*, *Lr19*, *Sr26*, and *Sr43* genes controlling rust resistance and the *Fhb7* gene controlling Fusarium head blight resistance from *Thinopyrum ponticum* and *Thinopyrum elongatum*, as well as powdery mildew resistance genes including *Pm21*, *Pm62*, and *Pm55* from *Dasypyrum villosum*, have been introgressed into wheat [13].

In order to guarantee future food and nutritional security, plant genetic resources are crucial [14]. Thus, evaluating genetic diversity and understanding the relationships between germplasm collections improves germplasm management and genetic advancement. Several anatomical and molecular attributes have been introduced for the evaluation of genetic variation in selected germplasm. The examination of divergence and grouping of individuals may be performed in a straightforward, precise, and expeditious way using genetic markers regarding their stability across tissues and growth stages, impenetrability by environmental effects, and fast techniques for declaring genetic variability [15]. The study of genetic diversity in native *Agropyron* spp. accessions is relevant for genetic resource conservation, widening the genetic base, and practical applications in breeding programs. Several molecular marker systems, such as simple sequence repeats (SSR) [4, 16], genotyping by sequencing (GBS) [17], and functional markers related to the P genome of Agropyron [18] have been used to evaluate the genetic variability of Agropyron spp. germplasm. Nowadays, because of the advent of molecular markers based on repeated sequences of the genome, such as ISSR markers [19], the genetic diversity of plant germplasm may be assessed in greater depth. These marker systems offer distinct advantages and have found widespread use in assessing genetic variation and population structure in forage crops [20].

Given the limited reports regarding the genetic variability of *Agropyron* spp. in Iran, it is hypothesized that significant genetic diversity will be exhibited by the domestic accessions of *Agropyron* spp. when they are assessed using ISSR markers. Furthermore, it is expected that distinct patterns in the population structure of these accessions will be revealed, which could contribute to the conservation of genetic resources and provide information for breeding programs concerning this important pasture plant.

## Materials and methods

### Plant material, genomic DNA extraction, and ISSR assay

In this study, 32 native accessions of *Agropyron* spp. from two species, *A*. *cristatum* (7 accessions) and *A*. *elongatum* (25 accessions), were generously provided by the Agricultural and Natural Resources Research and Education Center’s Gene Bank, Isfahan, Iran (https://esfahan.areeo.ac.ir/). Regarding the plant material, the collection of plant specimens adhered to the pertinent national guidelines and regulations. Table 1 shows the code and origin of the accessions investigated. Regarding Table 1, some accessions have the same code but differing numbers, for example, accessions 1000/116-1 and 1000/116-2, which show that this genotype originated from the common population. Because *Agropyron* is a cross-pollinated plant, each of the derived plants may have different characteristics. In the greenhouse, seedlings from each accession are grown in individual plastic pots. Once the plants had matured sufficiently, leaves from all of the pots were collected and combined to obtain genomic DNA. The genomic DNA was isolated using the technique described by Doyle and Doyle [21]. The DNA quality was determined by running 1μl DNA in 0.8% agarose (w/v) gels in 0.5X TBE buffer (45mM Tris base, 45mM boric acid, 1mM EDTA pH 8.0). In the next step, a NanoDrop spectrophotometer (ThermoFisher Scientific, USA) was used to measure the concentration of the DNA samples. Samples of DNA were then prepared for amplification by polymerase chain reaction (PCR) at a concentration of 20 ng/mol of genomic DNA. In this study, molecular diagnostics was applied by using 37 ISSR primers [22] presented in Table 2. For ISSR analysis, the polymerase chain reaction was carried out in a volume of 13 μl using the Biometra UNO II thermocycler (Analytik Jena, Germany). The reaction mixture contains 20 ng DNA, 6 μL master mix (dNTP, Mgcl_2,_ Taq DNA polymerase), 5 μL ddH2O, and 1 μL primer. The manufacturing of mater mixes and the synthesis of primers are accomplished by Sina Clone Company, Tehran, Iran. The PCR program was as follows: an initial step of 4 min at 94°C, followed by 35 cycles of 94°C for 45 s, 52°C to 54°C for 45 s (annealing temperature, which differed depending on the primer type), 72°C for 2 min, and 10 min at 72°C. The reaction products were mixed with an equal volume of formamide dye (98% formamide, 10 mM EDTA, 0.05% bromophenol blue, and 0.05% xylene cyanol) and resolved in 2% (w/v) agarose gel (0.5X TBE). Then, they were stained with ethidium bromide (1.0μg ml-1) and photographed under UV light by the Gel Documentation System (Bio-Rad, Canada). Here, all of the presented chemical reagents were prepared by Sigma-Aldrich.

**Table 1:** Membership percentage of each *Agropyron* accession to constructed subgroups (Q-matrix) as well as the genetic group name.

Accessions	Seed bank code	Species (ploidy level)	Q-matrix		Subgroup
			Q1	Q2	
**G01**	1000/116-1	*A*. *elangatum* (2n = 6x = 42)	0.171	0.829	Green
**G02**	1000/289-1		0.062	0.938	Green
**G03**	1000/251-1		0.002	0.998	Green
**G04**	1000/279-1		0.002	0.998	Green
**G05**	1000/429		0.033	0.967	Green
**G06**	1000/116-2		0.001	0.999	Green
**G07**	1000/279-2		0.002	0.998	Green
**G08**	1000/c12-1		0.002	0.998	Green
**G09**	1000/c11-1		0.001	0.999	Green
**G10**	1000/251-2		0.002	0.998	Green
**G11**	1000/281		0.003	0.997	Green
**G12**	1000/195		0.001	0.999	Green
**G13**	1000/196-1		0.002	0.998	Green
**G14**	1000/289-2		0.003	0.997	Green
**G15**	1000/279-3		0.003	0.997	Green
**G16**	1000/c11-2		0.313	0.687	Mix
**G17**	1000/289-3		0.298	0.702	Green
**G18**	1000/305		0.235	0.765	Green
**G19**	1000/289-4		0.351	0.649	Mix
**G20**	1000/c3		0.326	0.674	Mix
**G21**	1000/279-4		0.374	0.626	Mix
**G22**	1000/344		0.372	0.628	Mix
**G23**	1000/c11-3		0.45	0.55	Mix
**G24**	1000/196-2		0.434	0.566	Mix
**G25**	1000/c12-2		0.479	0.521	Mix
**G26**	1000/377	*A*. *cristatum* (2n = 4x = 28)	0.991	0.009	Red
**G27**	1000/388		0.998	0.002	Red
**G28**	1000/279		0.999	0.001	Red
**G29**	1000/62		0.999	0.001	Red
**G30**	1000/443		0.993	0.007	Red
**G31**	1000/102		0.996	0.004	Red
**G32**	1000/360		0.931	0.069	Red

**Table 2:** ISSR primer names, sequences, and amplification characteristics in the studied *Agropyron* panel.

Primer	Sequence (5ˊ- 3ˊ)	Annealing Temperature (˚C)	*A*. *elangatum*			*A*. *cristatum*		
			Total loci	Polymorphic loci	%polymorphism	Total loci	Polymorphic loci	%polymorphism
**ISSR 1**	(CA)_8_RG	52	13	13	100	13	12	92.3
**ISSR 2**	(GA)_8_YT	52	5	5	100	16	16	100
**ISSR 3**	(GA)_8_YG	52	16	16	100	6	6	100
**ISSR 4**	(AG)_8_YC	52	39	39	100	21	20	95.2
**ISSR 5**	(AG)_8_T	52	21	21	100	15	14	93.3
**ISSR 6**	(GA)_8_T	52	30	30	100	26	21	80.7
**ISSR 7**	(GA)_8_A	52	16	16	100	12	12	100
**ISSR 8**	CCC (GT)_7_	52	18	18	100	10	10	100
**ISSR 9**	(CA)_8_A	52	23	23	100	12	12	100
**ISSR 10**	(GT)_8_T	52	37	37	100	22	22	100
**ISSR 11**	(TC)_8_C	52	17	17	100	17	12	70.5
**ISSR 12**	(AG)_8_CC	52	47	47	100	15	6	40
**ISSR 13**	(AG)_8_YA	52	24	23	95.8	17	12	70.5
**ISSR 14**	(GT)_8_YA	52	32	32	100	26	25	96.1
**ISSR 15**	(AC)_8_YT	52	19	14	73.68	13	13	100
**ISSR 16**	(AC)_8_YA	52	30	30	100	22	19	86.3
**ISSR 17**	(AC)_8_YG	52	38	37	97.3	33	29	87.8
**ISSR 18**	(GACA)_4_	52	34	34	100	30	30	100
**ISSR 19**	(GGAGA)_3_	52	18	18	100	9	3	33.3
**ISSR 20**	(GA)_8_YA	52	34	34	100	28	28	100
**ISSR 21**	(GA)_8_RC	52	36	35	97.2	21	13	61.9
**ISSR 22**	(GGGGT)_3_	52	36	36	100	30	27	90
**ISSR 23**	AC)_8_GCT)	52	58	58	100	41	41	100
**ISSR 24**	(AC)_8_TG	52	40	38	95	27	27	100
**ISSR 25**	(TCC)_5_TG	52	28	28	100	24	14	58.3
**ISSR 26**	(AC)_8_GT	52	27	27	100	26	25	96.1
**ISSR 27**	(AG)_8_TC	52	29	29	100	19	17	89.4
**ISSR 28**	GA)_8_GCC)	52	35	35	100	25	21	84
**ISSR 29**	ACT ACG ACT (TG)_7_	52	10	9	90	13	7	53.8
**ISSR 30**	ACT CGT ACT (AG)_7_	52	10	10	100	9	6	66.6
**ISSR 31**	CGT AGT CGT (CA)_7_	52	21	18	85.7	17	13	76.4
**ISSR 32**	AGT CGT AGT (AC)_7_	52	10	7	70	10	5	50
**ISSR 33**	(GA)_9_C	54	26	25	96.1	21	21	100
**ISSR 34**	(CT)_9_G	56	27	25	92.5	20	17	85
**ISSR 35**	(CA)_10_G	54	14	7	50	15	6	40
**ISSR 36**	GT (CAC) _7_	57	29	29	100	17	12	70.5
**ISSR 37**	GT(CAC) _7_	57	9	4	44.4	7	0	0

### Data analysis

A binary data matrix was constructed by assigning a value of 0 (absence) or 1 (presence) to each band in the PCR amplification products for each of the 32 accessions. The POPGENE version 1.31 software [23] (University of Alberta, Canada) was then used to compute a variety of statistics, including the total number of loci, the number of polymorphic loci, the effective number of alleles (ne), the Shannon’s Information Index (I) [24], and the Nei’s genetic diversity (h) [25]. A model-based Bayesian approach in the program STRUCTURE 2.3.4 [26] (University of Stanford, USA) was used to infer population structure among the examined *Agropyron* spp. accessions. In this regard, 10 independent runs were performed with the setting the number of subpopulations (K) from 1 to 10, burn in time and MCMC (Markov Chain Monte Carlo) replication numbers both set to 500,000, and a model for admixture and correlated allele frequencies. Delta K (ΔK) based on the second-order rate of change in the likelihood [27] was used to represent the K value. Inferred ancestry estimates of individuals (Q matrix) were derived for the selected population [28]. In order to classify and visualize specimen dispersion, the principal coordinate analysis as well as neighbor-joining clustering were implemented by GenAlEx [29] and Darwin (CIRAD, France) software, respectively. The linkage disequilibrium (LD) was estimated with TASSEL 2.1 [30] (Cornell University, USA). In this study, Jaccard similarity coefficients were calculated among accessions belonging to each species separately. Then, using a similarity matrix, the UPGMA algorithm has been applied to classify accessions of *A*. *cristatum* as well as *A*. *elongatum* in Darwin software [31].

## Results

**Fig 1:**
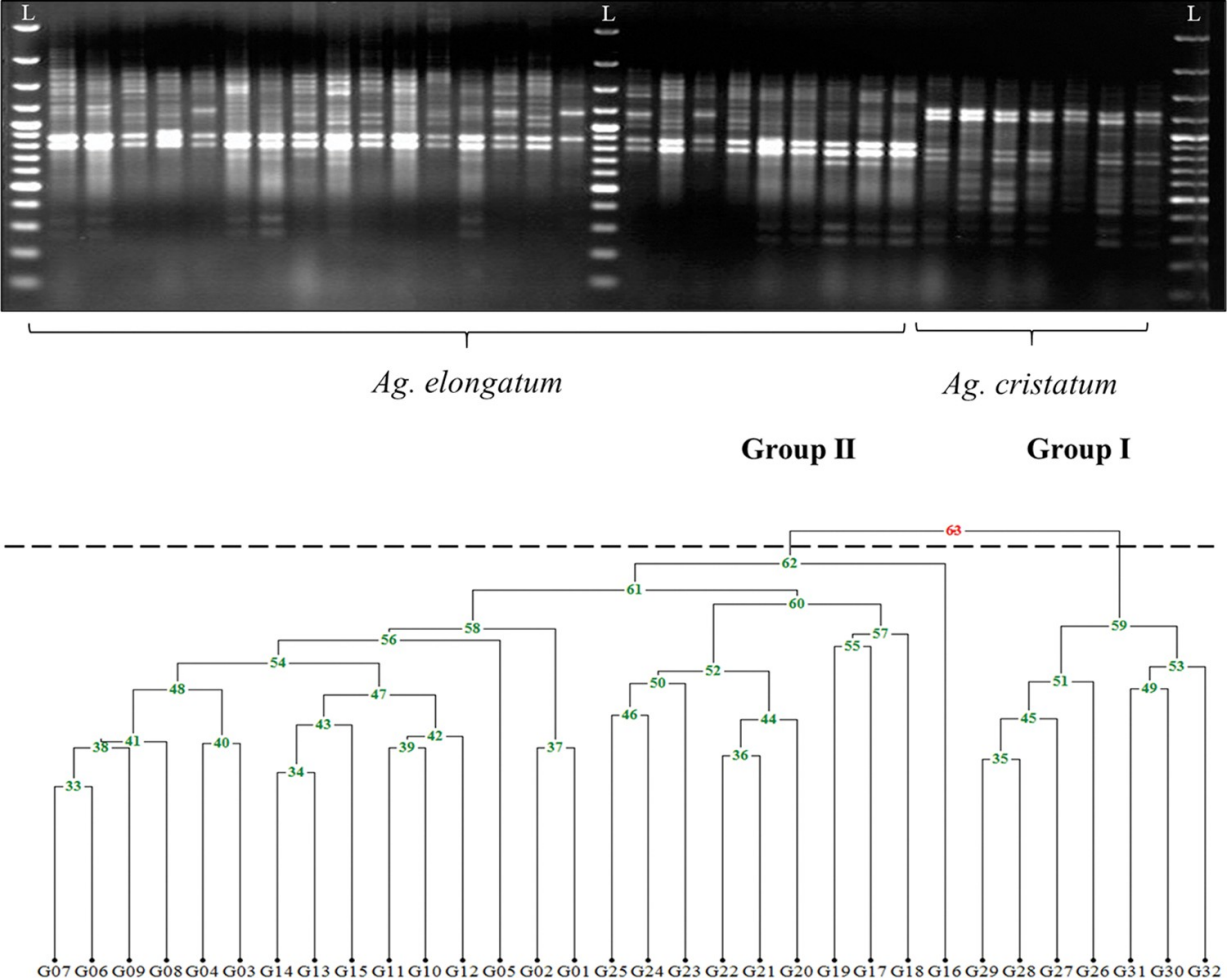
A: Genotyping profile of *Agropyron spp*. accessions using the "ISSR16" primer (the scoring district in gel was illustrated with a red box, and a 1kb DNA ladder was used in this study); B: Classification of the studied plant germplasm based on the UPGMA algorithm.

**Fig 2:**
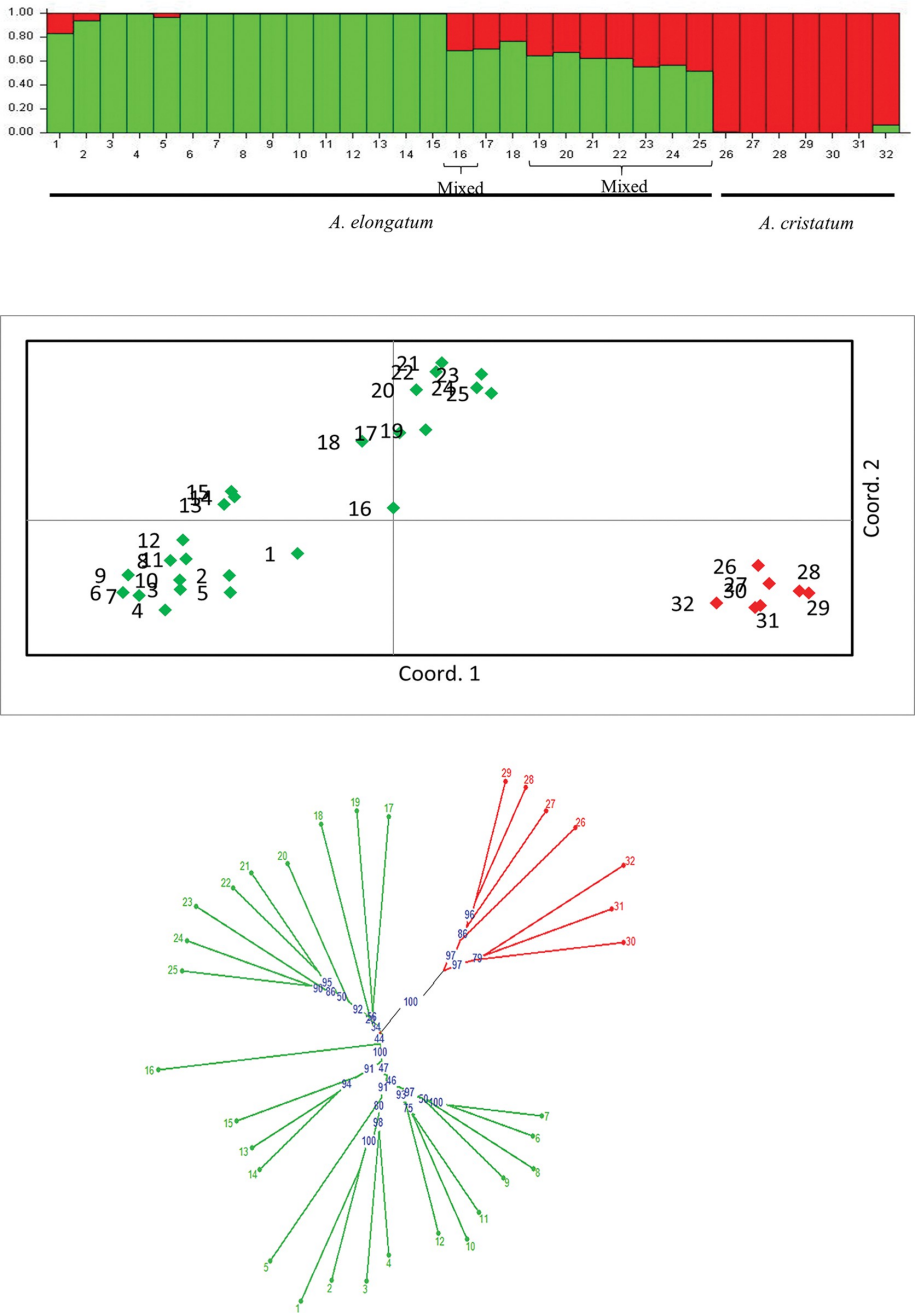
A: Genetic relatedness of studied *Agropyron* germplasm based on 1013 ISSR markers (The color of the bars indicates the two subpopulations identified through the STRUCTURE program); B: Principal coordinate analysis for visualization of *Agropyron* germplasm based on ISSR data; C: Classifying studied *Agropyron* genotypes based on identified ISSR loci using neighbour joining clustering method.

The molecular evidence presented in this study clearly demonstrates the presence of genetic diversity among the cultivated *Agropyron* accessions from Iran. The DNA marker analysis revealed a total of 956 loci in the genome of *A*. *elongatum* and 705 loci in the genome of *A*. *cristatum* (Table 2). A sample output of germplasm fingerprinting is shown in Fig 1A. In the species *A*. *elongatum*, the highest and lowest values of amplified loci were calculated for ISSR23 ((AC)_8_GCT) and ISSR2 ((GA)_8_YT) respectively. Also in the species *A*. *cristatum*, ISSR23 ((AC)_8_GCT) and ISSR3 ((GA)_8_YG) had the maximum and minimum values of total loci, respectively. Results showed that 97% of amplified loci in *A*. *elongatum* and 84% of amplified loci in *A*. *cristatum* are polymorphic (Table 2). Using the Jaccard similarity matrix and UPGMA algorithm, the studied *Agropyron* spp. accessions were partitioned into two main groups, including groups I and II (Fig 1B). Group I involved accessions that belonged to the species *A*. *cristatum*, while all accessions from the species *A*. *elongatum* were located in group II. Here, the highest value of the calculated cophenetic correlation coefficient (r = 0.93) verified the suitability of the distance measurement method and also the classification algorithm. A model-based Bayesian technique in the program STRUCTURE was utilized to reveal the genetic structure of the analyzed germplasm. After using Evanno’s approach, the most likely value of K was found to be two. Table 1 shows the membership percentage of each accession to recognized subgroups (Q values). Accordingly, 32 *Agropyron* spp. accessions were partitioned into two main subgroups, including “green” and “red” which corresponded to two species (Fig 2A–2C). Some accessions, including "G16", "G19", "G20", "G21", "G22", "G23", "G24" and "G25" were identified in the “Mix” subgroup (Table 1 and Fig 2A) in order to have Q values lower than %70 in the group [32] and therefore, these genotypes have a more likely admixture genome.

**Fig 3:**
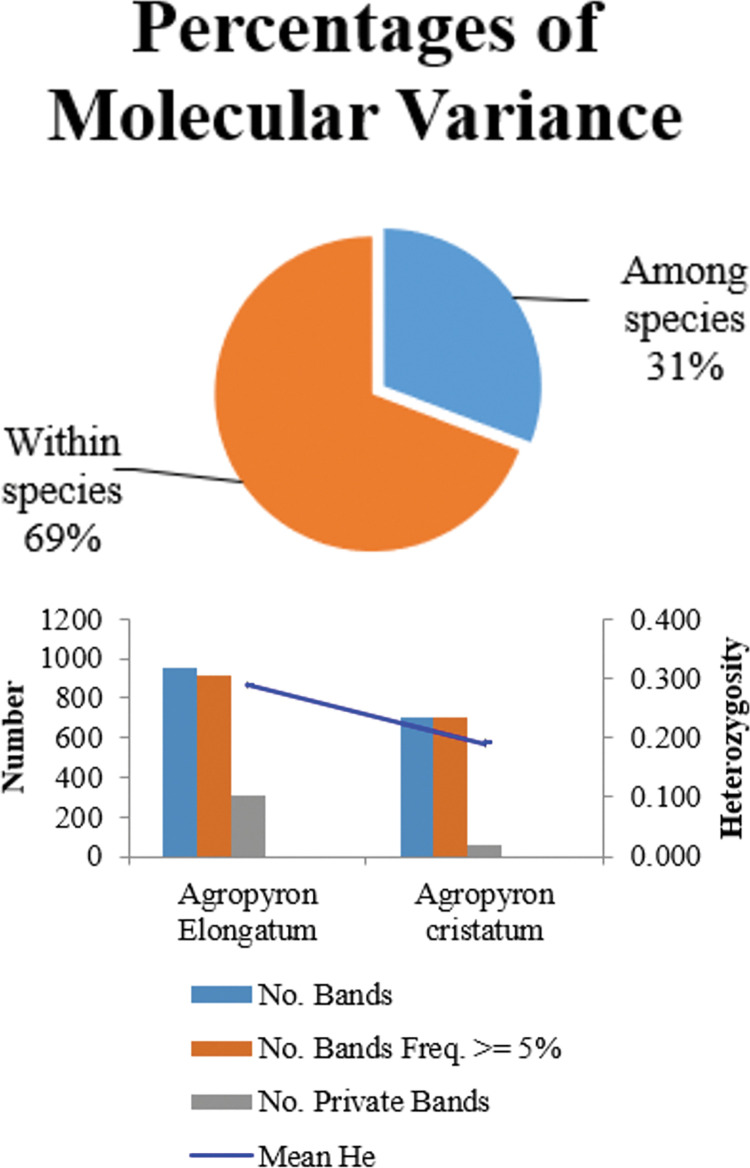
Analysis of molecular variance (A) and ISSR genotyping pattern across studied *Agropyron* species (B). No. Bands = No. of Different Bands, No. Bands Freq. > = 5% = No. of Different Bands with a Frequency > = 5%; No. Private Bands = No. of Bands Unique to a Single Population; He = Expected Heterozygosity = 2×p×q.

**Fig 4:**
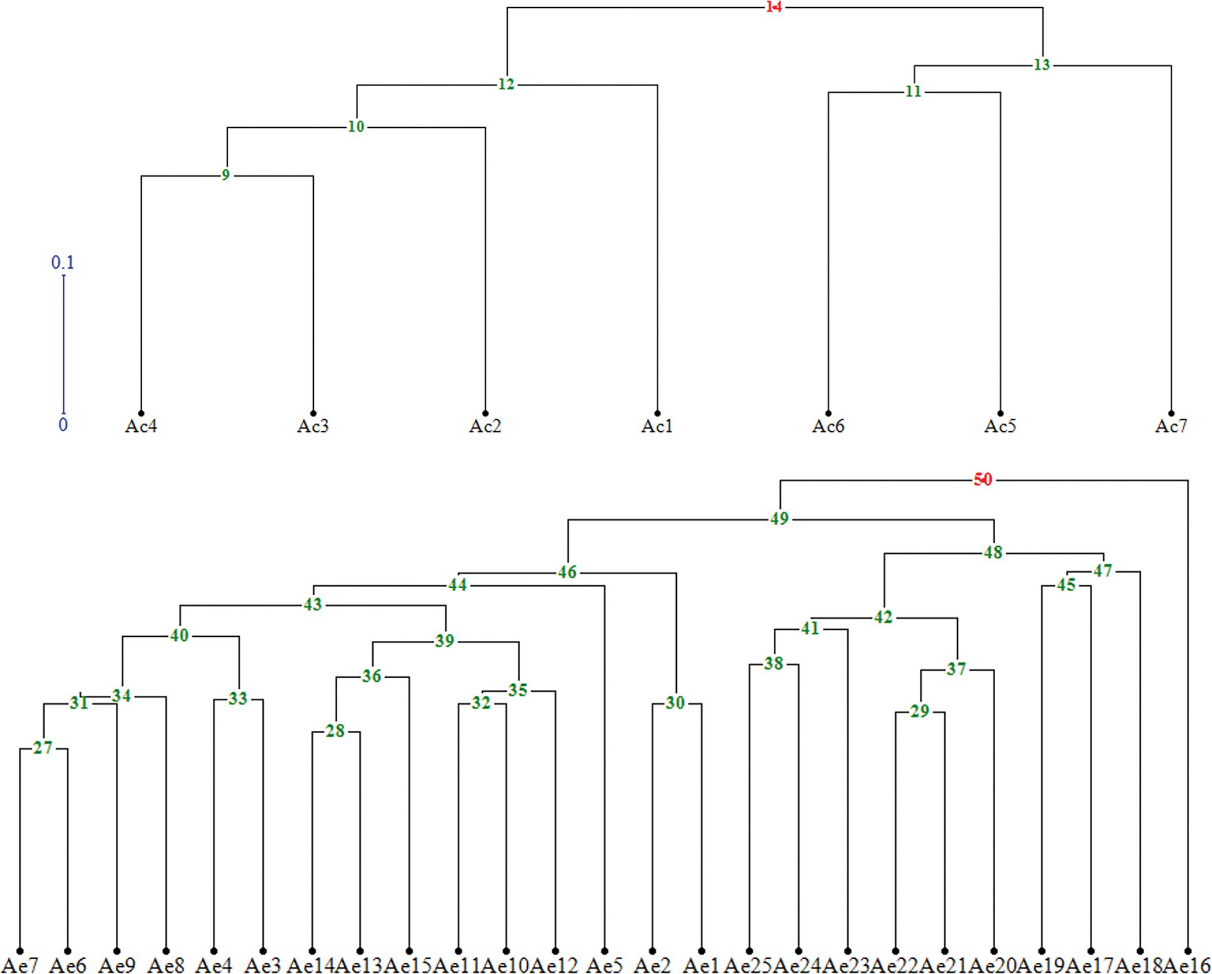
Identification of heterotic groups in studied *A*. *cristatum* (A) and *A*. *elongatum* (B) accessions using the UPGMA algorithm.

**Fig 5:**
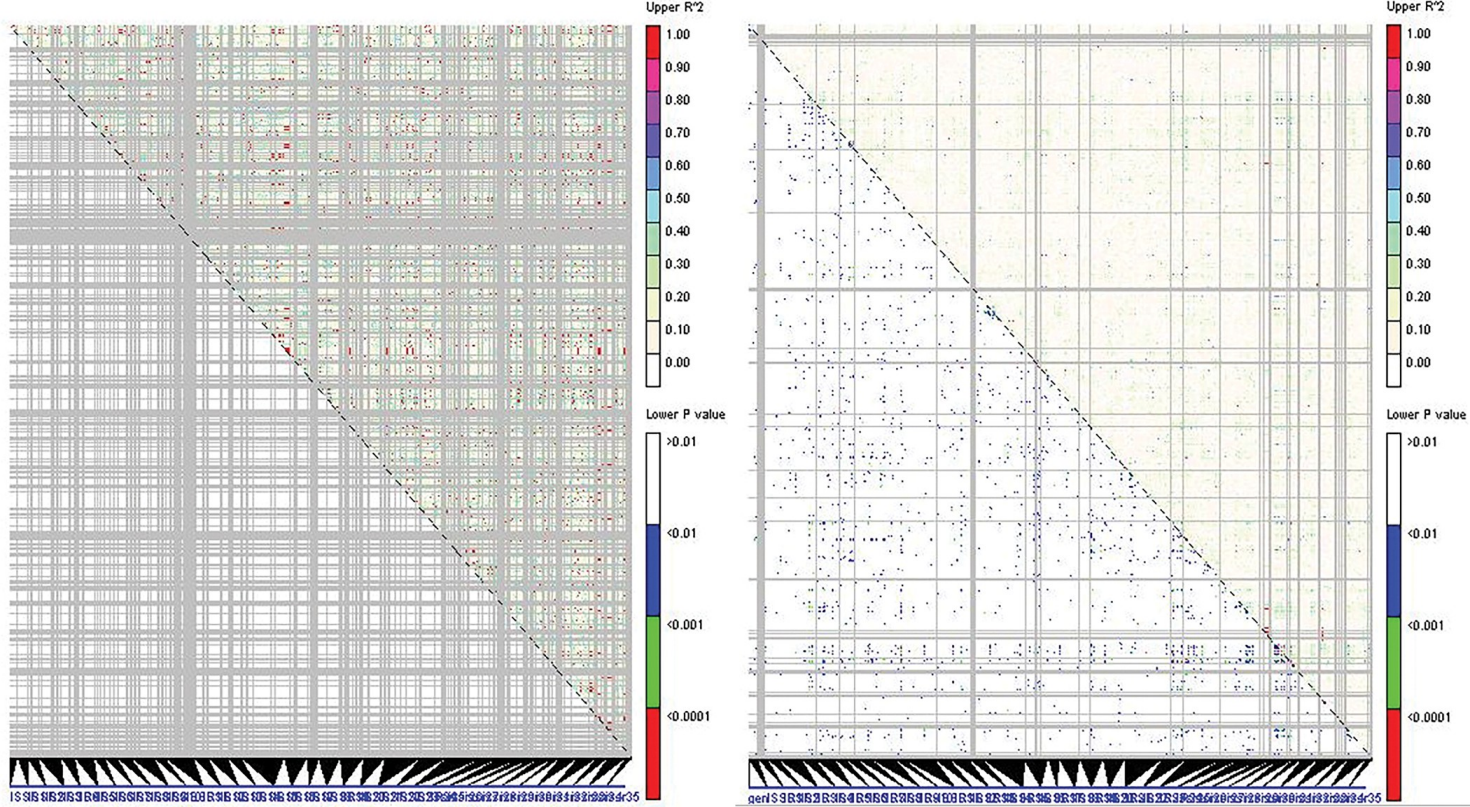
Linkage disequilibrium calculated for *A*. *cristatum* (A) and *A*. *elongatum* (B). above and below diagonals depict r^2^ and P-values, respectively.

Here, analysis of molecular variance by assuming the existence of two identified subgroups (Fig 3A), revealed that 31% of genetic variability belonged to between-groups (between species), whereas 69% of variation was related to within-groups (within species). As shown in Fig 3B and Table 3, the mean values of the Na, Ne, I and He parameters at *A*. *elongatum* were higher than those found at *A*. *cristatum* (Table 3). Also, the number of private loci detected for *A*. *elongatum* was higher than that for *A*. *cristatum* (Fig 3B). Regarding the r^2^ value [33] 5.2% and 0.5% of all possible pairs of ISSR loci showed a significant level of LD (P≤0.05) in *A*. *elongatum* and *A*. *cristatum*, respectively (Fig 4A and 4B). The high level of LD opens an avenue for studying genome-wide association analysis on this inspected association panel. In the following, the inspection of genetic variability inside each species was done by calculating Jaccard similarity coefficients (Tables 4 and 5) accompanied by the UPGMA clustering algorithm (Fig 5A and 5B). Overall, the Jaccard similarity coefficient had low values for both *Agropyron* species. In detail, the Jaccard similarity coefficient varied between 0.33 and 0.66 in species of *A*. *cristatum* (Table 4) and 0.25 and 0.7 in species of *A*. *elongatum* (Table 5). Moreover, two and three heterotic groups were identified for each of the *A*. *cristatum* (Fig 5A) and *A*. *elongatum* (Fig 5B) species, respectively. In the species *A*. *cristatum* (Fig 4A), three accessions were located in the same group, and others established a second group, whereas the arrangement of *A*. *elongatum* accessions in heterotic groups was as follows: three groups with one, nine, and fifteen accessions.

**Table 3:** Descriptive statistics of marker data per species and overall, where N is the number of samples, Na is the number of different alleles, Ne is the number of effective alleles, I is Shanon`s information index and He is the expected heterozygosity.

	Mean and standard error over loci for each species					
			Na	Ne	I	He
***A*. *elongatum***	Mean		1.857	1.476	0.440	0.288
	standard error		0.015	0.010	0.007	0.005
					
***A*. *cristatum***	Mean		1.282	1.317	0.289	0.190
	standard error		0.028	0.011	0.009	0.006
	Grand mean and standard error over loci and species					
		N	Na	Ne	I	He
	Mean	16.000	1.570	1.397	0.364	0.239
	standard error	0.200	0.017	0.008	0.006	0.004

**Table 4:** Jaccard similarity coefficients between individuals of *A*. *cristatum*.

	Ac1	Ac2	Ac3	Ac4	Ac5	Ac6	Ac7
**Ac1**	1.00						
**Ac2**	0.55	1.00					
**Ac3**	0.50	0.61	1.00				
**Ac4**	0.52	0.56	0.66	1.00			
**Ac5**	0.42	0.45	0.46	0.52	1.00		
**Ac6**	0.39	0.46	0.47	0.51	0.53	1.00	
**Ac7**	0.33	0.34	0.39	0.41	0.47	0.52	1.00

**Table 5:** Jaccard similarity coefficients between individuals of *A*. *elongatum*.

	Ae1	Ae2	Ae3	Ae4	Ae5	Ae6	Ae7	Ae8	Ae9	Ae10	Ae11	Ae12	Ae13	Ae14	Ae15	Ae16	Ae17	Ae18	Ae19	Ae20	Ae21	Ae22	Ae23	Ae24	Ae25
**Ae1**	1.00																								
**Ae2**	0.64	1.00																							
**Ae3**	0.49	0.57	1.00																						
**Ae4**	0.49	0.57	0.63	1.00																					
**Ae5**	0.39	0.43	0.49	0.53	1.00																				
**Ae6**	0.46	0.51	0.53	0.61	0.52	1.00																			
**Ae7**	0.42	0.48	0.51	0.58	0.48	0.70	1.00																		
**Ae8**	0.39	0.48	0.48	0.56	0.43	0.60	0.64	1.00																	
**Ae9**	0.41	0.49	0.47	0.56	0.46	0.62	0.65	0.63	1.00																
**Ae10**	0.37	0.42	0.44	0.48	0.43	0.54	0.51	0.50	0.59	1.00															
**Ae11**	0.37	0.44	0.44	0.50	0.44	0.56	0.55	0.52	0.62	0.64	1.00														
**Ae12**	0.36	0.42	0.44	0.49	0.43	0.51	0.52	0.51	0.59	0.62	0.62	1.00													
**Ae13**	0.36	0.41	0.42	0.46	0.42	0.48	0.46	0.48	0.52	0.54	0.58	0.61	1.00												
**Ae14**	0.34	0.39	0.43	0.47	0.40	0.47	0.45	0.48	0.49	0.52	0.54	0.57	0.68	1.00											
**Ae15**	0.36	0.42	0.44	0.45	0.38	0.47	0.47	0.45	0.47	0.49	0.51	0.55	0.57	0.62	1.00										
**Ae16**	0.25	0.28	0.32	0.32	0.31	0.32	0.31	0.32	0.32	0.34	0.33	0.35	0.36	0.37	0.38	1.00									
**Ae17**	0.30	0.33	0.32	0.34	0.28	0.33	0.34	0.35	0.37	0.38	0.38	0.42	0.44	0.45	0.46	0.39	1.00								
**Ae18**	0.36	0.39	0.40	0.37	0.31	0.41	0.40	0.40	0.44	0.37	0.42	0.42	0.41	0.41	0.44	0.32	0.44	1.00							
**Ae19**	0.32	0.34	0.33	0.35	0.31	0.35	0.34	0.36	0.37	0.35	0.38	0.41	0.40	0.40	0.40	0.31	0.46	0.44	1.00						
**Ae20**	0.38	0.39	0.37	0.39	0.32	0.40	0.41	0.42	0.44	0.39	0.43	0.42	0.44	0.42	0.44	0.30	0.44	0.50	0.52	1.00					
**Ae21**	0.36	0.38	0.33	0.37	0.30	0.38	0.37	0.40	0.42	0.36	0.40	0.39	0.43	0.43	0.42	0.27	0.41	0.43	0.43	0.62	1.00				
**Ae22**	0.37	0.39	0.33	0.36	0.29	0.41	0.38	0.41	0.41	0.35	0.40	0.37	0.40	0.42	0.39	0.27	0.40	0.41	0.41	0.56	0.65	1.00			
**Ae23**	0.33	0.36	0.31	0.34	0.28	0.36	0.34	0.37	0.35	0.31	0.35	0.34	0.37	0.36	0.35	0.25	0.37	0.39	0.38	0.48	0.53	0.57	1.00		
**Ae24**	0.34	0.35	0.34	0.35	0.32	0.37	0.36	0.37	0.36	0.30	0.35	0.35	0.36	0.36	0.35	0.28	0.35	0.41	0.35	0.47	0.50	0.51	0.55	1.00	
**Ae25**	0.33	0.35	0.32	0.35	0.29	0.37	0.36	0.37	0.36	0.31	0.36	0.37	0.38	0.37	0.38	0.28	0.35	0.36	0.38	0.47	0.50	0.50	0.51	0.58	1.00

## Discussion

Beside the feeding application of the genus *Agropyron*, some species, such as *A*. *cristatum* [34] and *A*. *elongatum* [3] possess promising novel genes that can be used in wheat improvement. Iran is considered a rich resource for *Agropyron* spp. [35], and thus collection and diversity analysis of domesticated *Agropyron* accessions are critical in order to accelerate wheat breeding programs and achieve sustainable agriculture through rangeland management. In this study, 32 domesticated *Agropyron* accessions from the species *Agropyron elongatum* and *Agropyron cristatum* were examined. Our finding showed the existence of genetic variability among accessions based on ISSR markers, which was in line with reports by Mohammadi, Panahi [36] and Mohammadi, Amiri [20]. As inferred by the represented ISSR assay, the genetic variability among *Agropyron* spp. accessions can be inferred and clarified by focusing on the genomic sequence between two SSRs. Also, similar to previous reports [20, 37] each studied ISSR primer could amplify numerous loci across the genome of *Agropyron* spp. and so this marker system could be implemented for the construction or saturation of the genetic linkage map of *Agropyron* spp. [38] in addition to diversity analysis. Interestingly, the majority of amplified loci in two species were polymorphic, and this item may result from the old history of the genus *Agropyron* and the accumulation of mutation and genetic recombination within this period. In this research, the highest value of amplified loci achieved by primer ISSR23 ((AC)_8_GCT) could be effectively applied for the evaluation of genetic variability among *Agropyron* accessions.

The categorization of the examined accessions, employing both the UPGMA method and Bayesian analysis, unveiled the presence of two distinct groups, with each group comprising accessions belonging to the same species. So, it can be exploited that these species (*A*. *elongatum* and *A*. *cristatum*) have different genetic structures. Hence, it is mandatory to exert population structure and kinship relationships in association mapping studies of *Agropyron* spp. to avoid false positive results in identifying significant trait-marker relationships [39]. It is concluded that by applying ISSR markers, a breeder can distinguish two species from each other at the primary growth stage and save time. *Agropyron* spp. differentiation is important because each species has its own growth habit; for example, *A*. *elongatum* has a longer days to flowering period as well as a shorter plant height than *A*. *cristatum* [4, 40]. In the following, an ANOVA for ISSR data manifested the magnitude of within-species genetic variability, which was paralleled with the findings of Che, Yang [16] and Absattar, Absattarova [2]. Here, Nei [25] and Shannon [24] diversity indices also verified a high level of diversity within *A*. *elongatum* from Iran. Among the studied *Agropyron* species, *A*. *elongatum* had more private alleles than *A*. *cristatum*, which implies promising selection potential in *A*. *elongatum*. Evidence of private alleles in both analyzed accessions demonstrates their evolutionary importance [41]. On the other hand, both studied species have notable conservation value except for *A*. *elongatum*, which has a higher proportion. The population structure, a tool employed to elucidate the connections between individuals within and among populations [28], offered valuable insights into the evolutionary relationships among the individuals studied within the provided germplasm. As a benefit of this method, genotypes with an admixture genome could be recognized. For instance, in the present study, in addition to the identified subgroups (Green and Red), eight accessions ("G16", "G19", "G20", "G21", "G22", "G23", "G24" and "G25") are admixtures. Such mixed accessions may be a consequence of selection pressure or seed transfer between regions [42]. However, the cross-pollinated nature of *Agropyron* spp. could also lead to this phenomenon. Close to population structure, the existence of LD is a prerequisite for a given plant population to be utilized in association mapping analysis [33]. In this study, two studied species had different sample sizes, but generally, the LD value belonged to the species *A*. *elongatum* was higher than the LD value calculated for *A*. *cristatum*. Such a level of LD could guarantee the success of marker-trait association identification through an association mapping approach in *Agropyron* spp. [17] especially *A*. *elongatum*.

These additional analyses, involving the calculation of Jaccard similarity coefficients and species-specific classification, were undertaken to pinpoint unique genotypes within each species. This is crucial because the genetic distance between parental genotypes stands as the foremost factor influencing the manifestation of the heterosis phenomenon [43]. Here, the calculated Jaccard coefficients between accessions inside each species had lower values, implying a vast within-species diversity. So, it is possible to achieve genetic progress through simple selection in *Agropyron* germplasm. Likewise, identified genotypes from different heterotic groups can be implemented for the production of synthetic varieties, which is the best breeding method for forage crops like *Agropyron* spp. [44] to achieve heterosis.

## Conclusion

In summary, the genetic diversity within *Agropyron* species from Iran, particularly *A*. *elongatum* and *A*. *cristatum*, stands out significantly, especially in the context of their utilization as fodder crops. This germplasm has been effectively characterized and differentiated using ISSR markers. Both of the studied *Agropyron* species exhibit distinct historical evolutionary patterns, as demonstrated through population structure analysis. Our research underscores the presence of substantial genetic diversity and the identification of distant heterotic groups within each of these species, which underscores the potential for leveraging heterosis in the development of forage cultivars. Additionally, concerning linkage disequilibrium, our study suggests that the studied *Agropyron* panel, as well as other panels from Iran, holds promising potential for application in genome-wide association studies aimed at mapping specific desirable traits.

## Supporting information

S1 Raw images (PDF)
