# Supplementary material for: Molecular evidence depicts genetic divergence among Agropyron elongatum and A. cristatum accessions from gene pool of Iran
Source: PLoS One. 2023 Nov 30;18(11):e0294694. doi: 10.1371/journal.pone.0294694 (PMC10688953; doi:10.1371/journal.pone.0294694)

Genotyping profiles of Agropyron spp. accessions were determined using ISSR primers. In all the gel images presented below, from left to right: Lanes 1, 18, and 35 correspond to the DNA 1kb ladder, Lanes 2-17 and 19-27 represent samples of A. elongatum, and Lanes 28-34 correspond to samples of A. cristatum.

Samples of ISSR gels:

ISSR 1 (top) ISSR 2 (bottom)

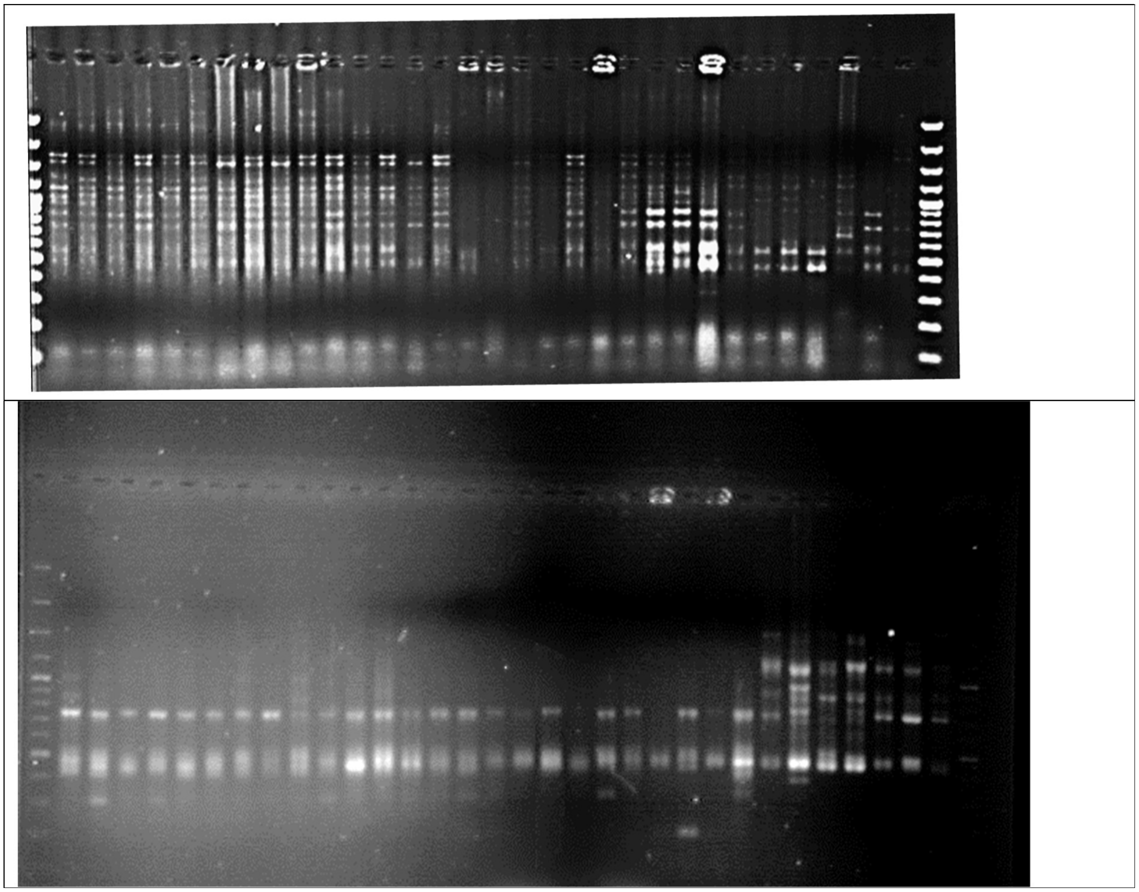

ISSR 3 (top) ISSR 4 (bottom)

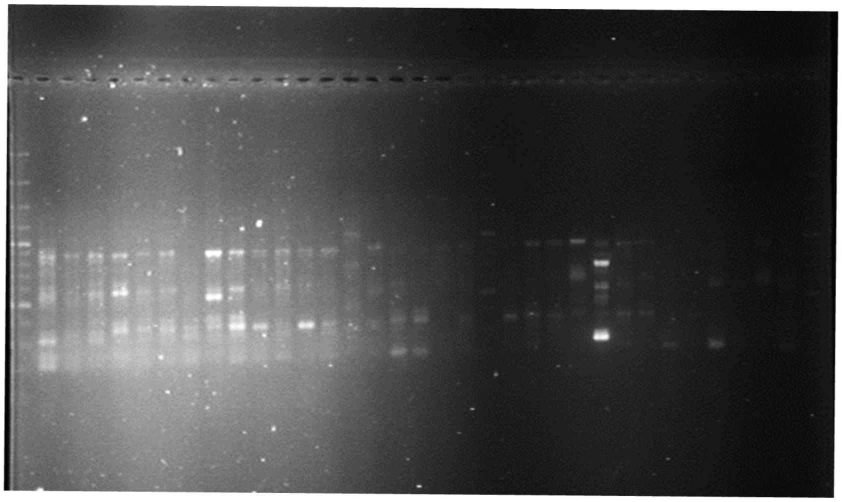

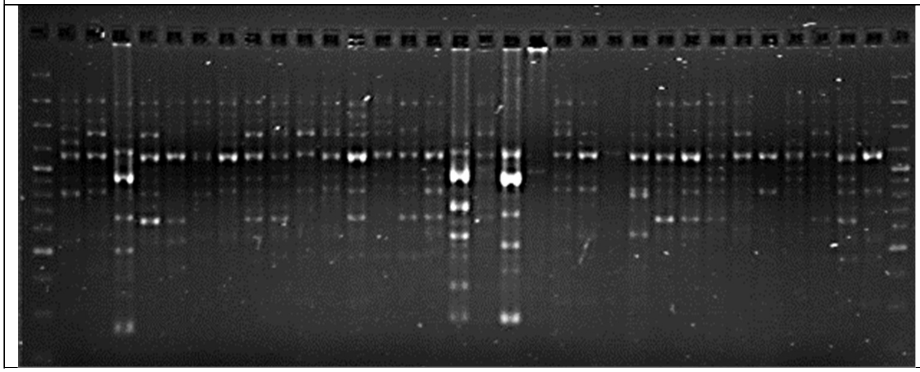

ISSR 5 (top) ISSR 6 (bottom)

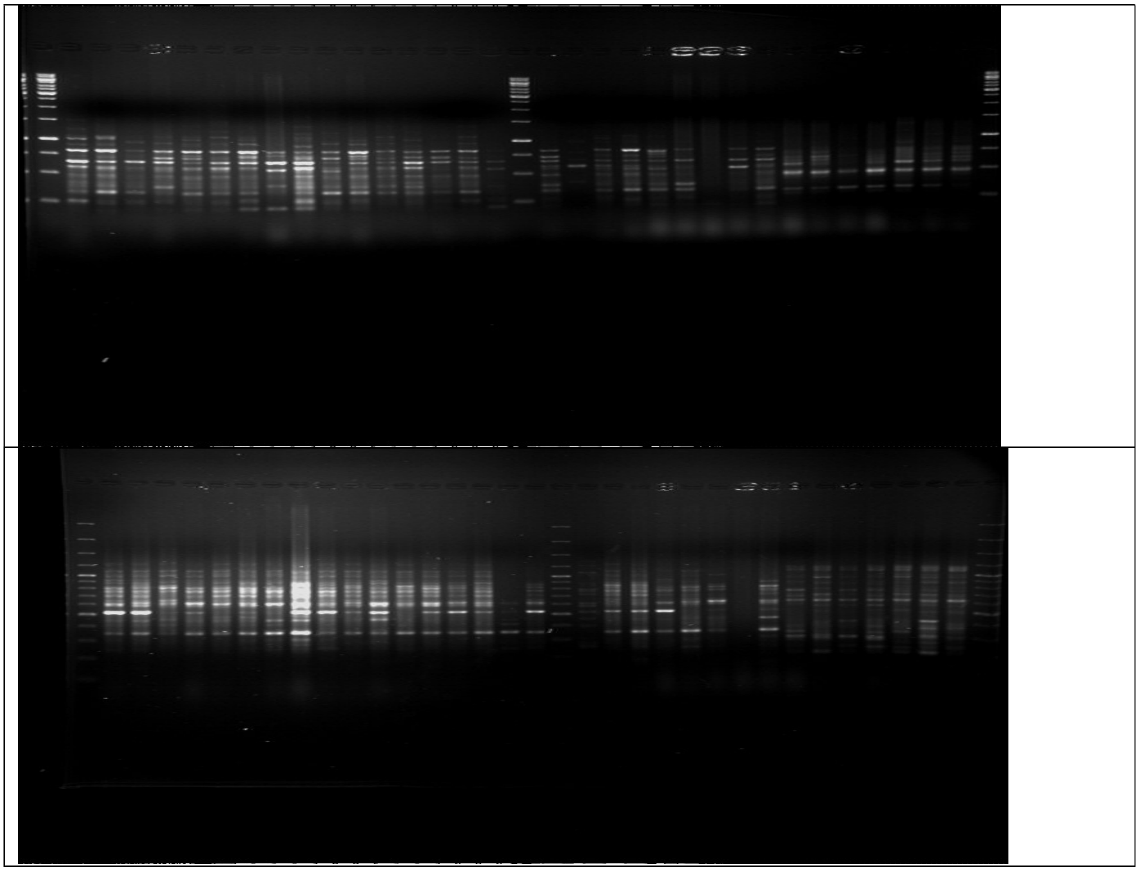

ISSR 7 (top) ISSR 8 (bottom)

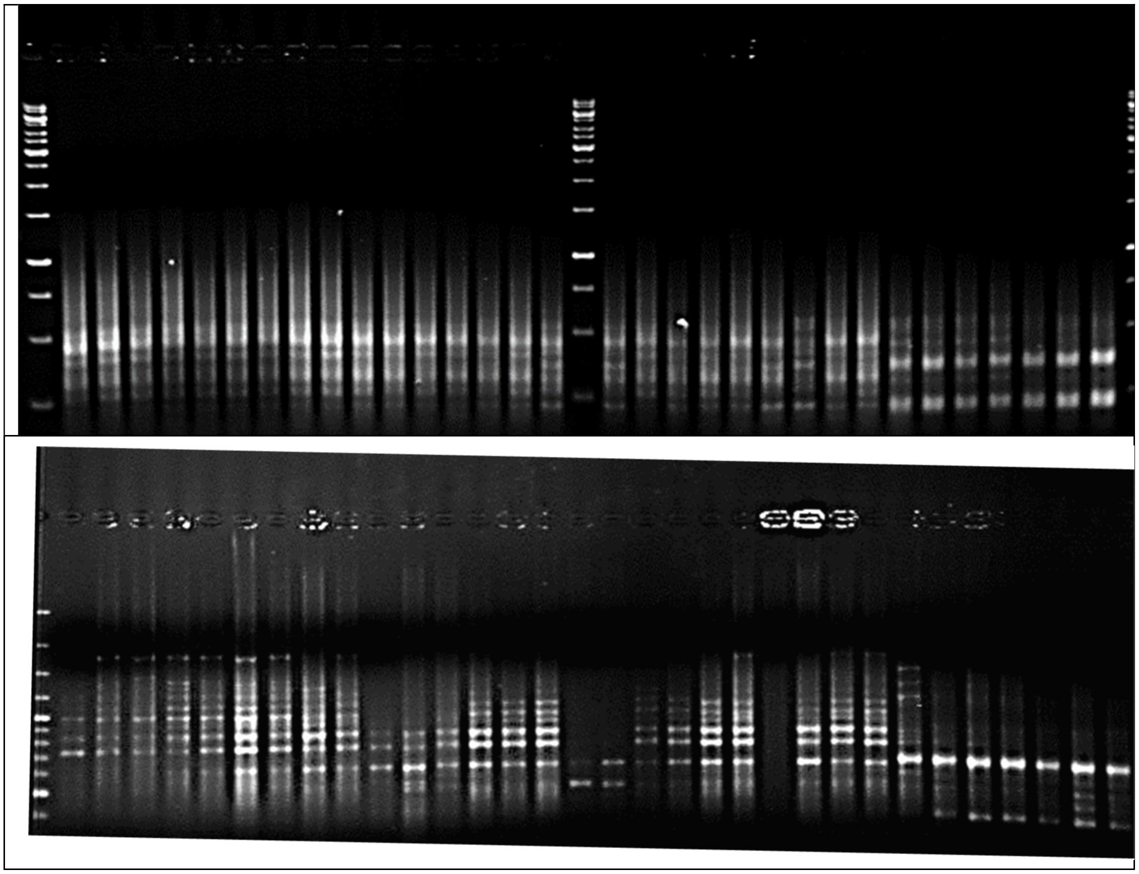

ISSR 9 (top) ISSR 10 (bottom)

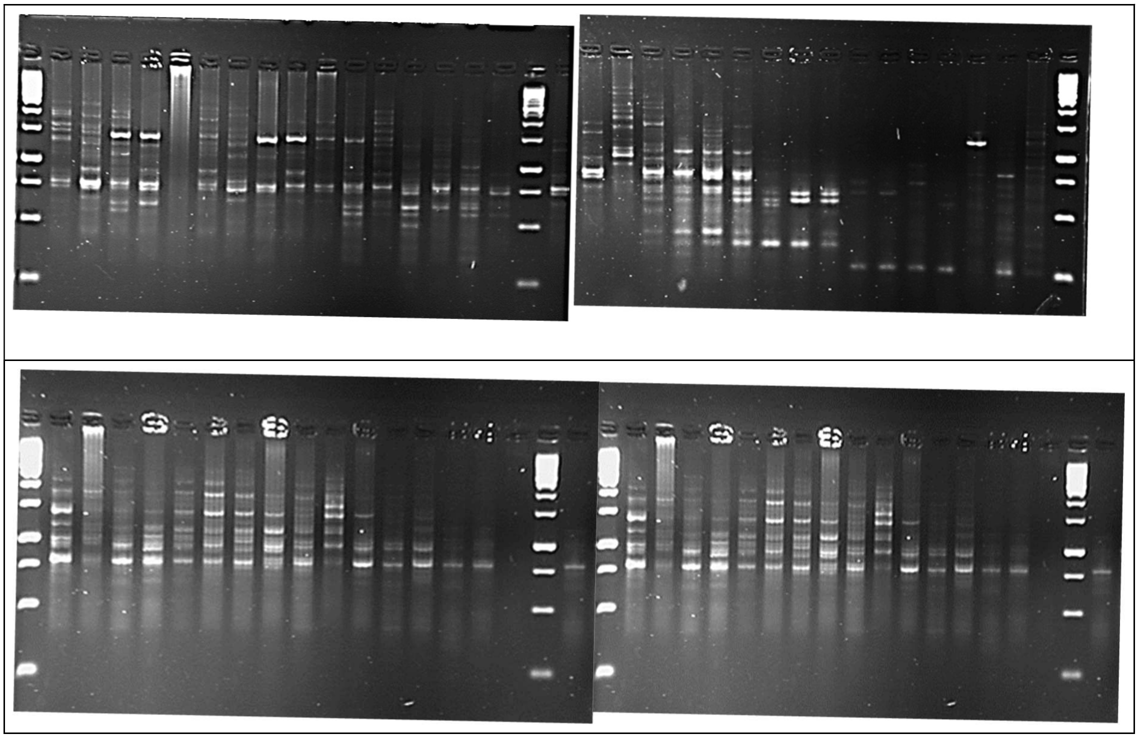

ISSR 11 (top) ISSR 12 (bottom)

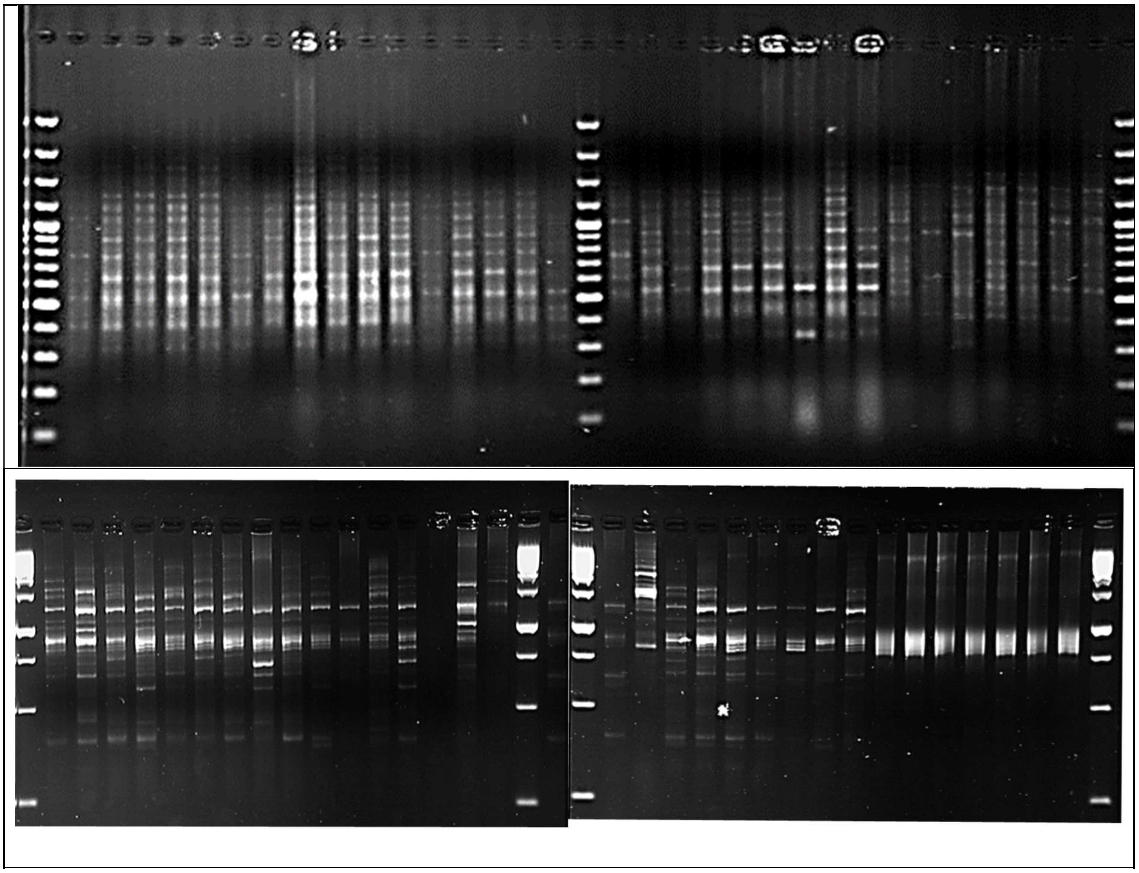

ISSR 13 (top) ISSR 14 (bottom)

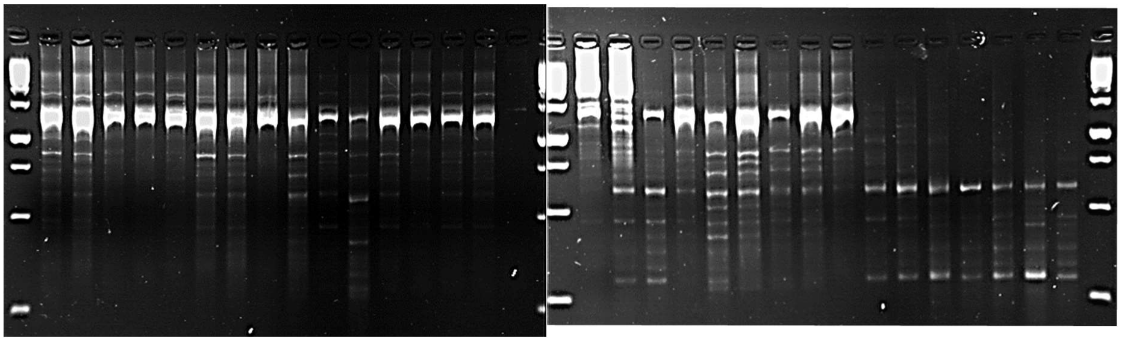

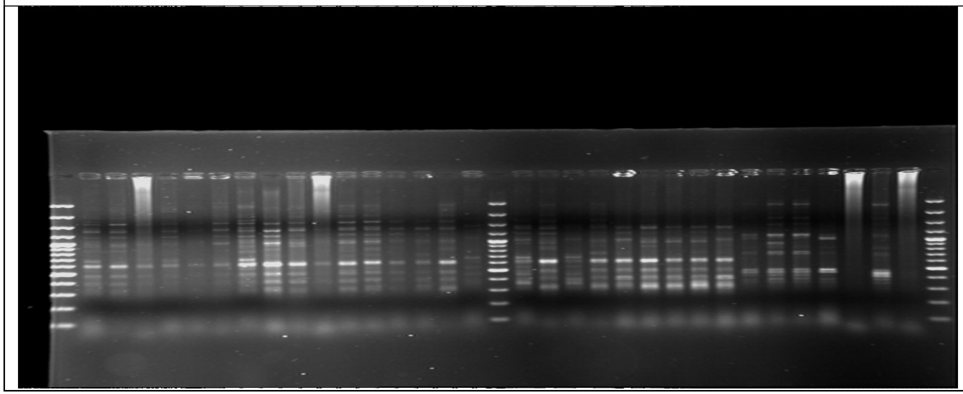

ISSR 15 (top) ISSR 16 (bottom)

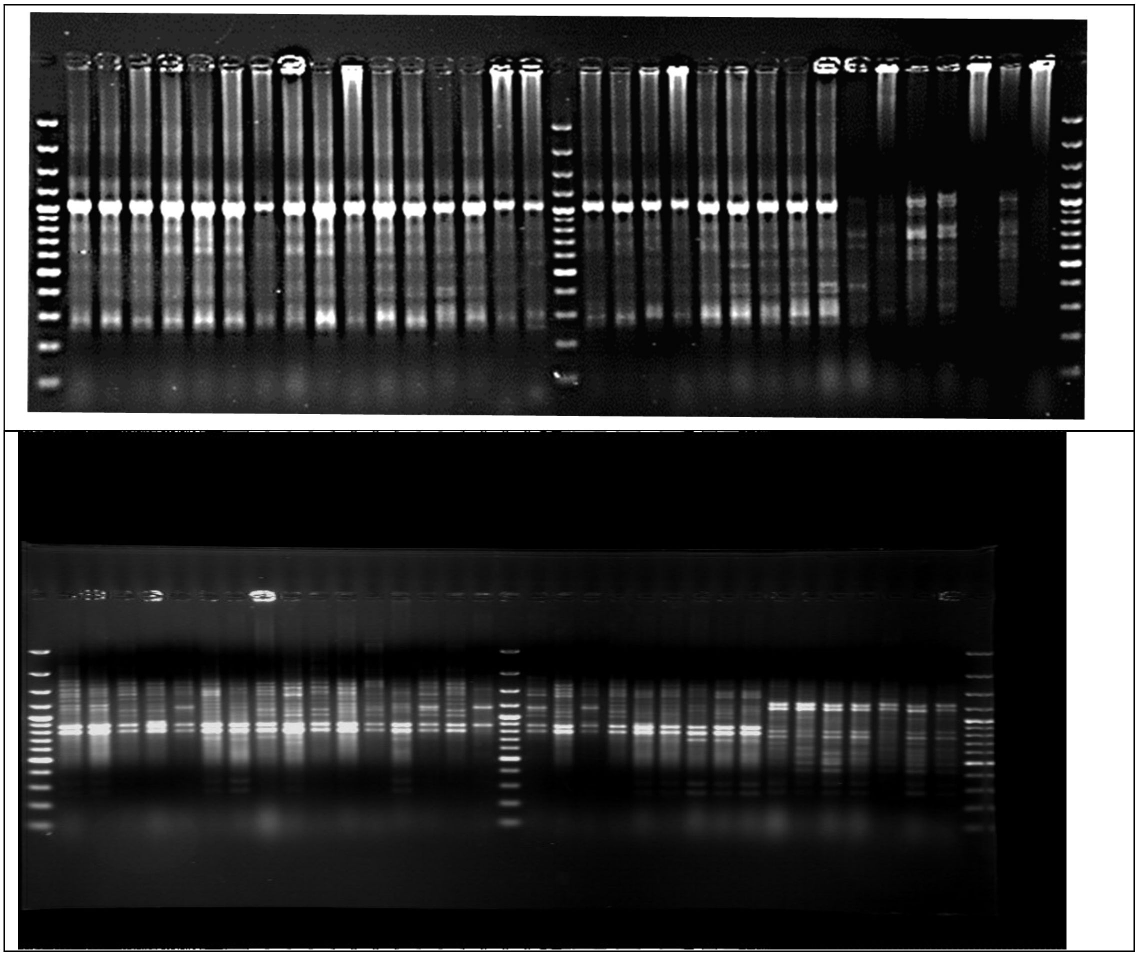

ISSR 17 (top) ISSR 18 (bottom)

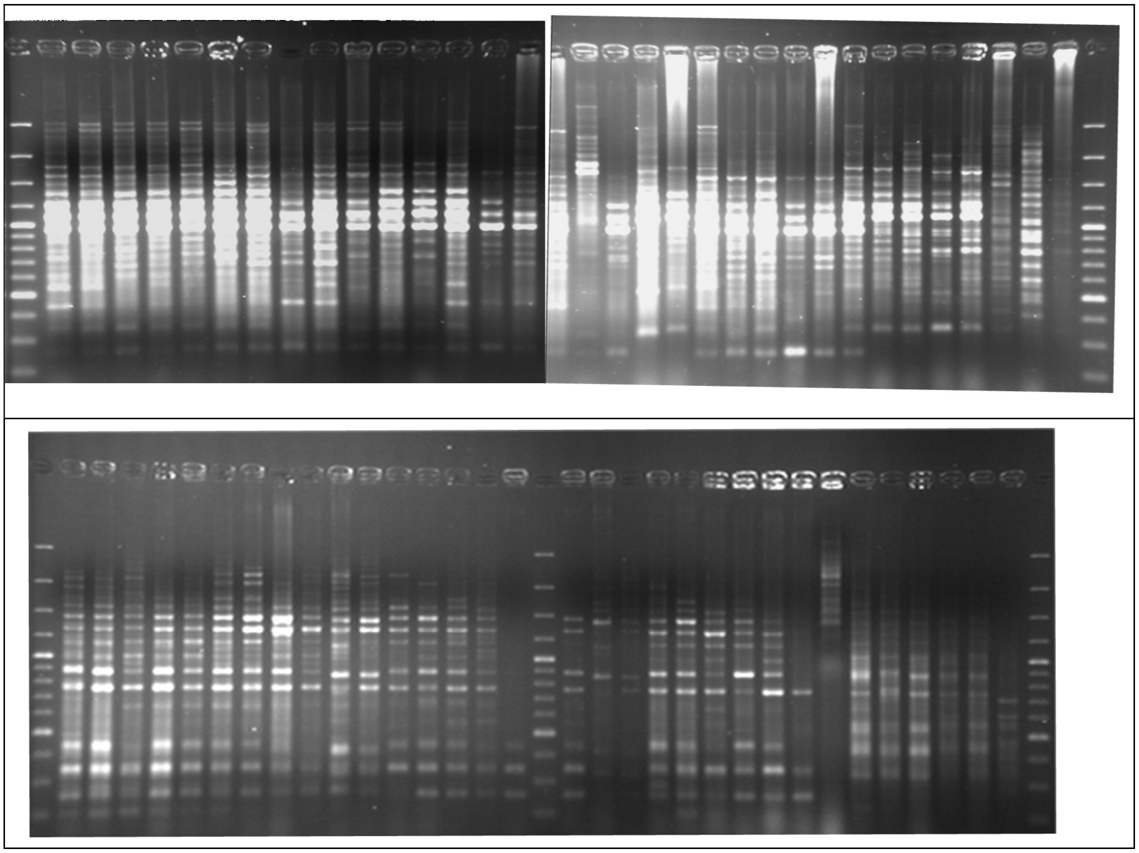

ISSR 19 (top) ISSR 20 (bottom)

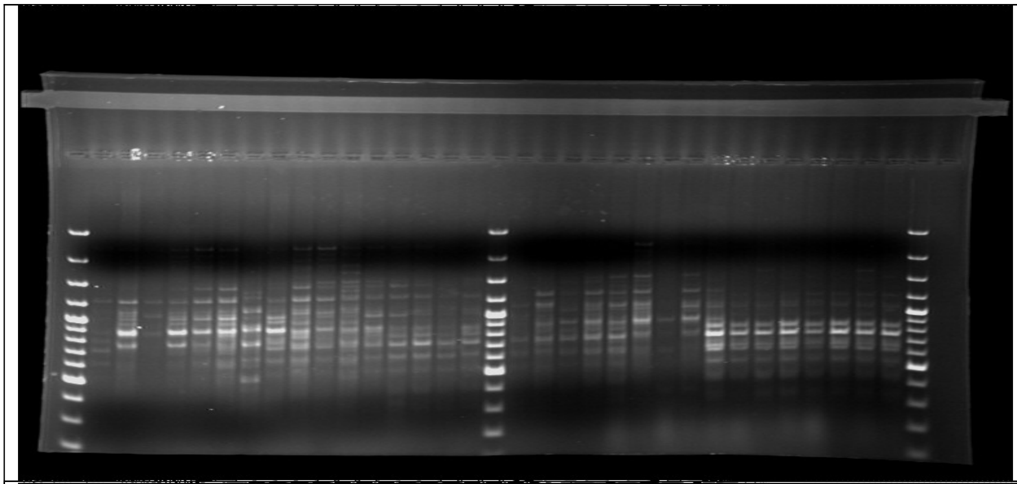

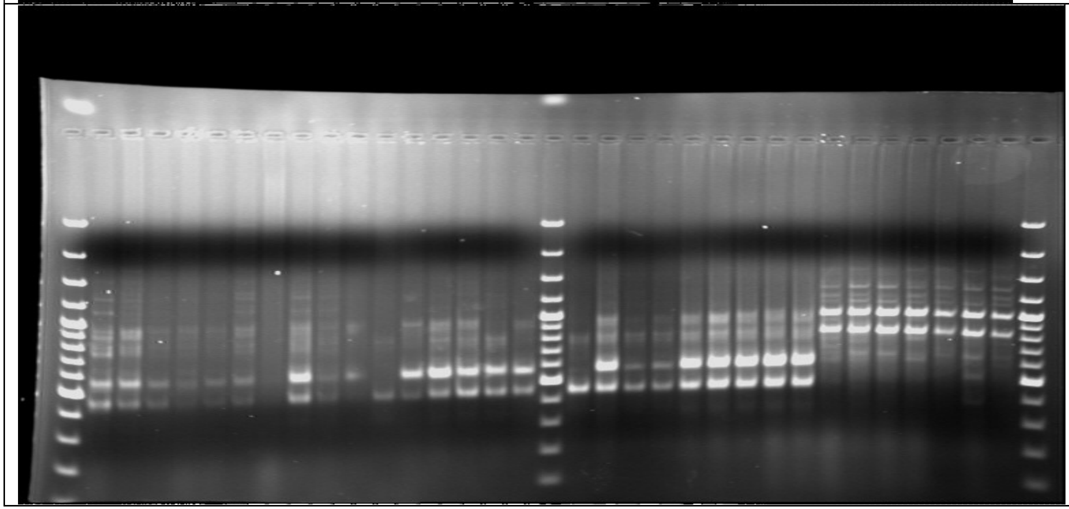

ISSR 21 (top) ISSR 22 (bottom)

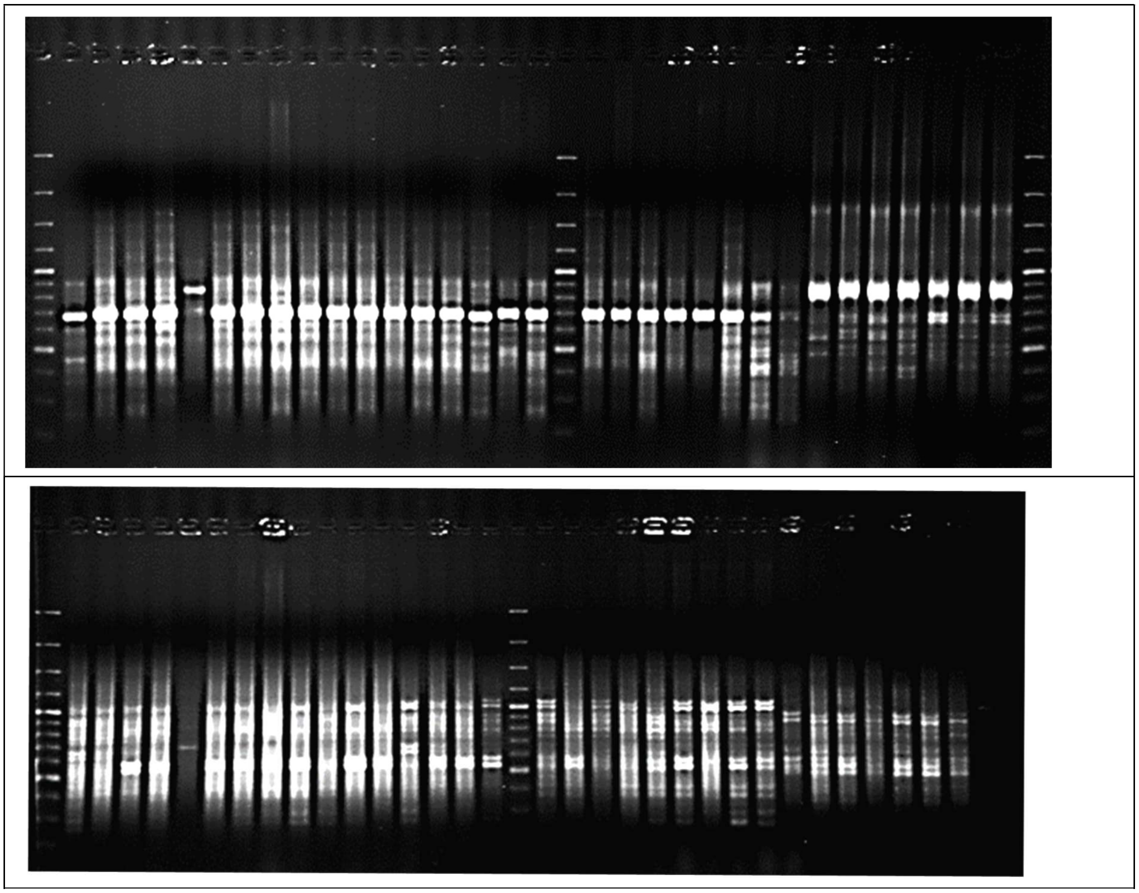

ISSR 23 (top) ISSR 24 (bottom)

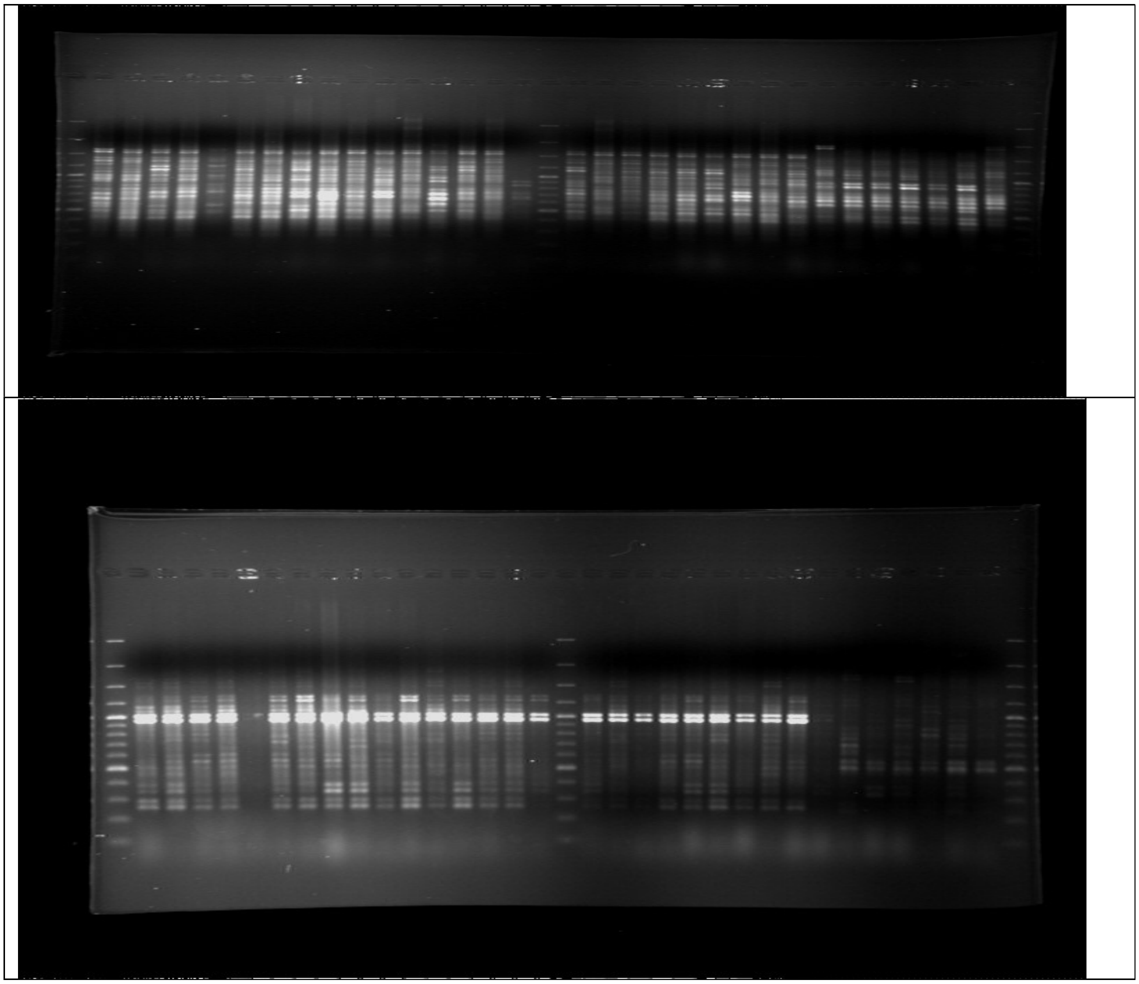

ISSR 25 (top) ISSR 26 (bottom)

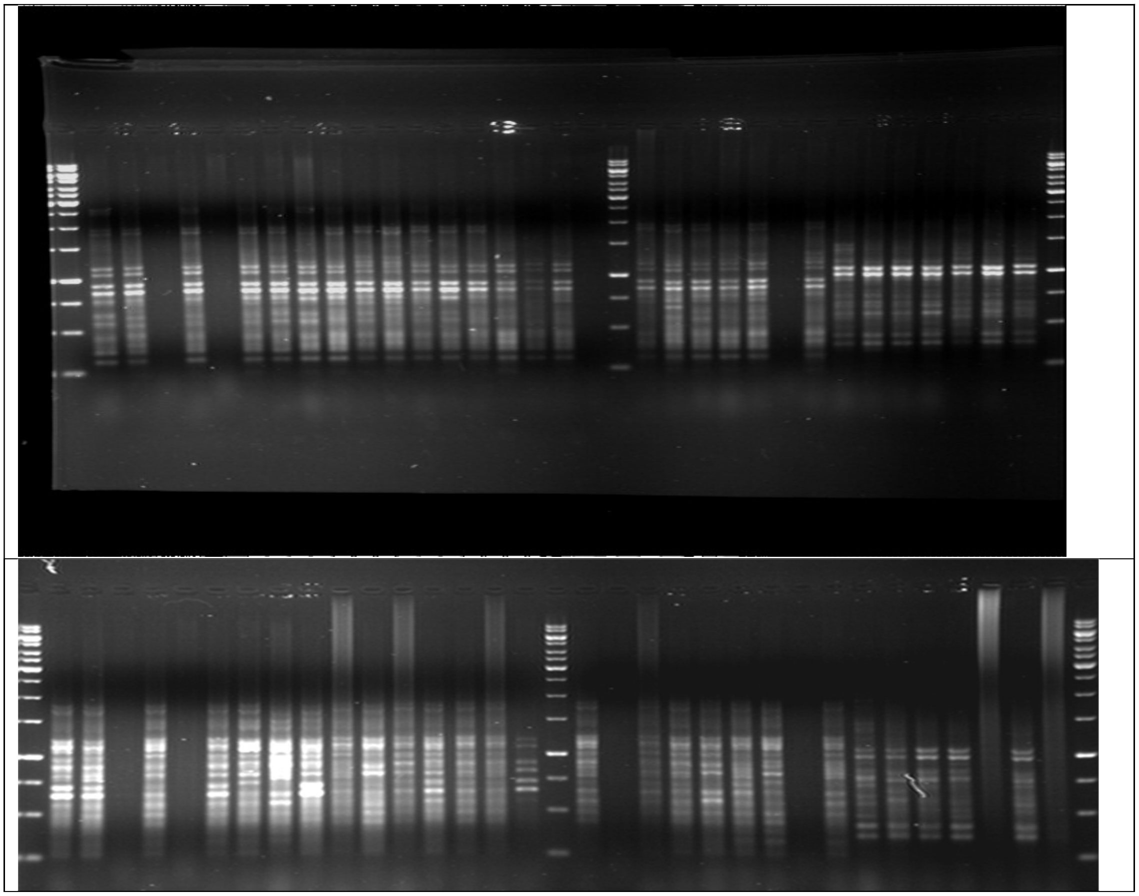

ISSR 27 (top) ISSR 28 (bottom)

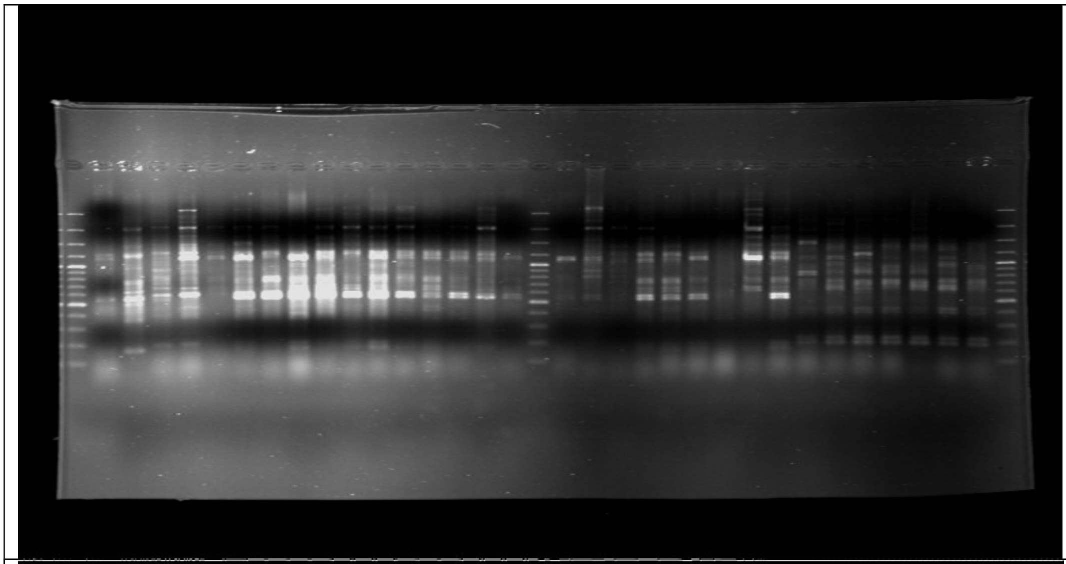

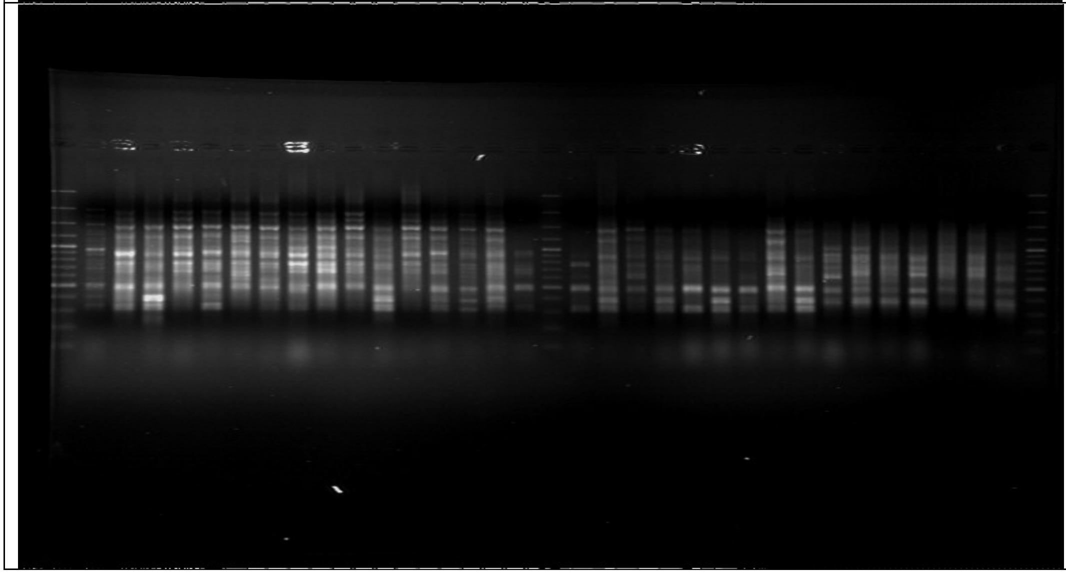

ISSR 29 (top) ISSR 30 (bottom)

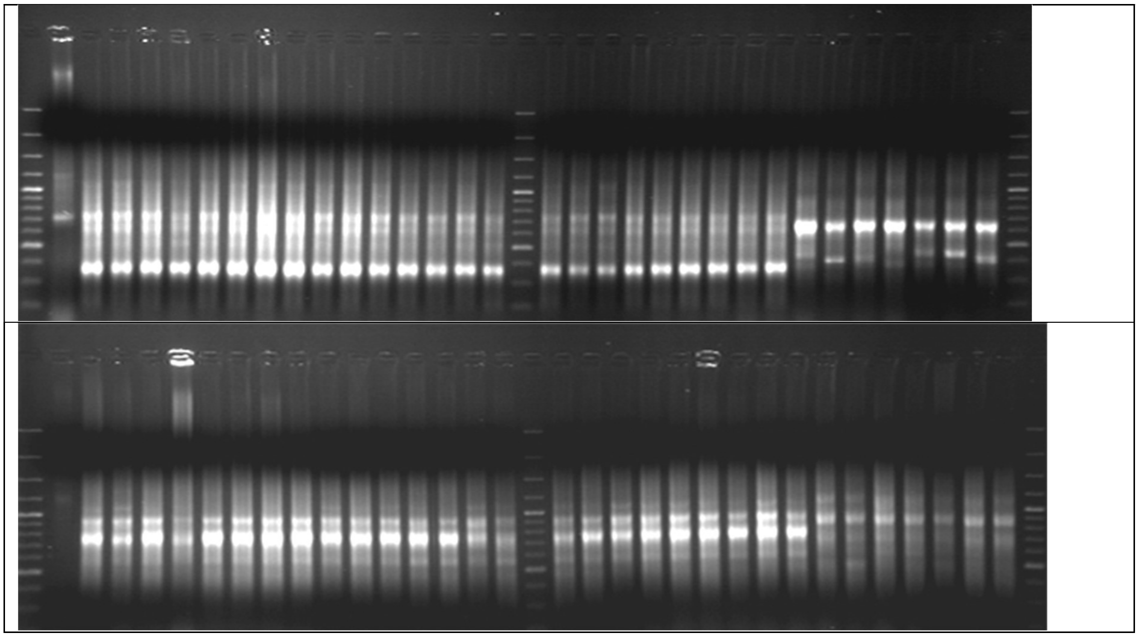

ISSR 31 (top) ISSR 32 (bottom)

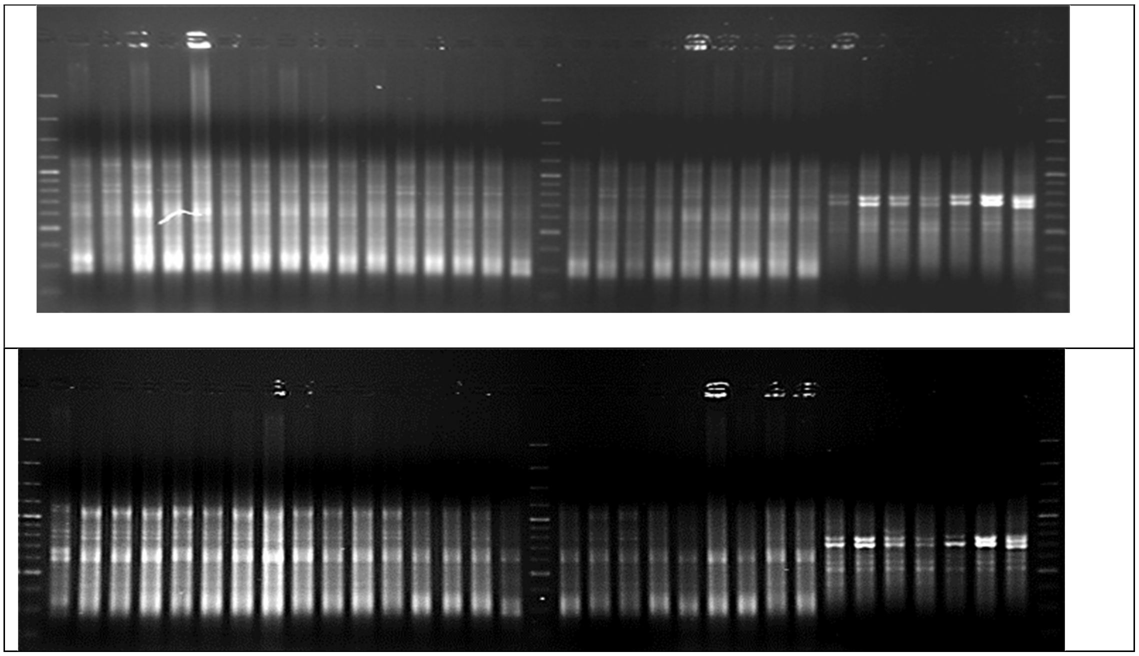

ISSR 33 (top) ISSR 34 (bottom)

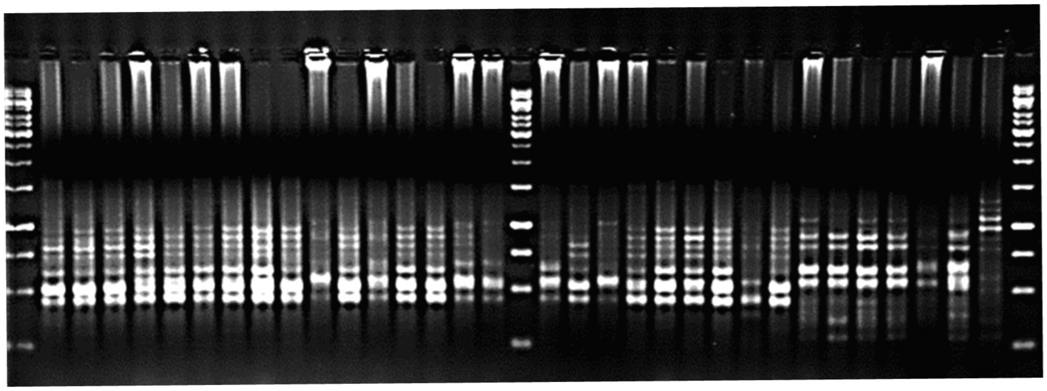

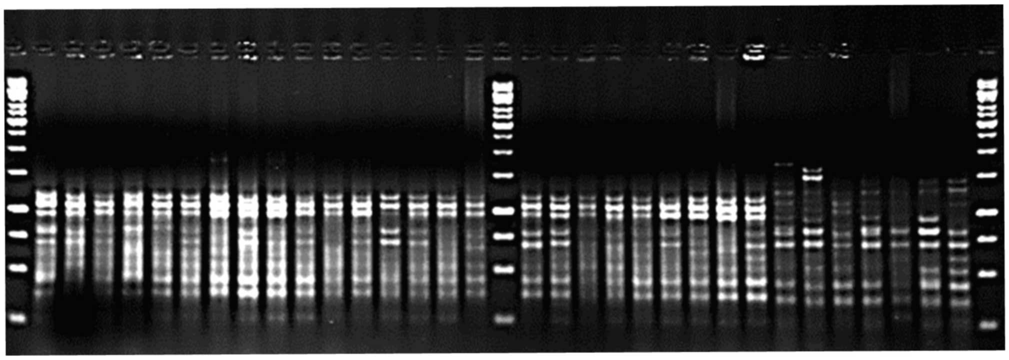

ISSR 35 (top) ISSR 36 (bottom)

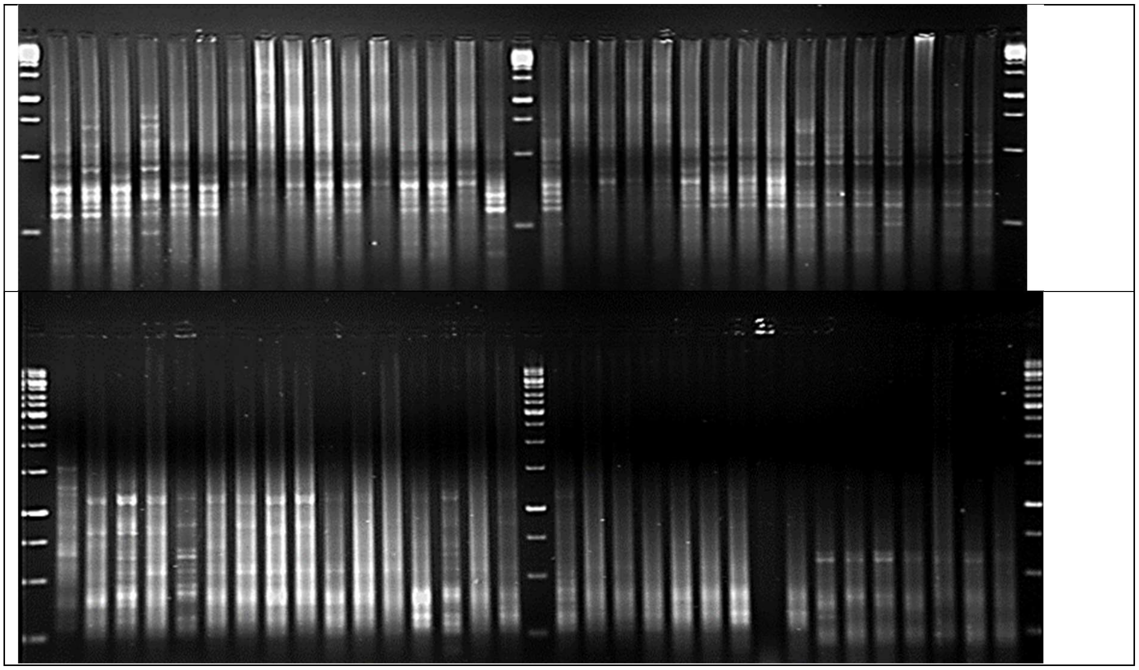

ISSR 37

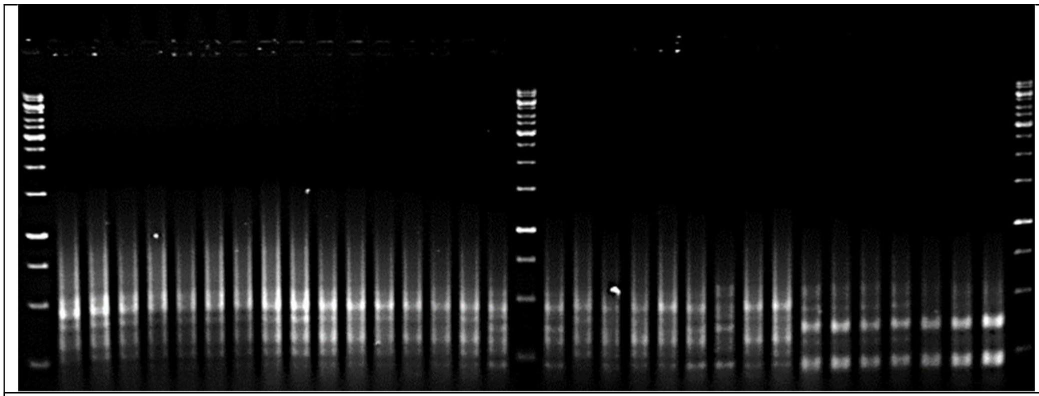

Supplement: S1 Raw images — (PDF) [file pone.0294694.s001.pdf]
